# Conventional MRI/CT for differentiation of IDH-mutant adult-type diffuse gliomas: revisited with a new imaging feature “scFLAIR-D”

**DOI:** 10.1007/s11604-026-01965-z

**Published:** 2026-03-27

**Authors:** Yuji Ohizumi, Ryo Kurokawa, Yusuke Watanabe, Mariko Kurokawa, Yosuke Kitagawa, Masashi Nomura, Hirokazu Takami, Nobuhito Saito, Osamu Abe

**Affiliations:** 1 Department of Radiology, Graduate School of Medicine, The University of Tokyo, 7-3-1 Hongo, Bunkyo-ku, Tokyo, 113-8655 Japan; 2 Department of Neurosurgery, Graduate School of Medicine, The University of Tokyo, 7-3-1 Hongo, Bunkyo-ku, Tokyo, 113-8655 Japan

**Keywords:** World Health Organization, Astrocytoma, Oligodendroglioma, T2-FLAIR mismatch, Magnetic resonance imaging, Computed tomography

## Abstract

**Purpose:**

To test whether a refined definition of the T2–FLAIR mismatch sign (redefined T2FM) and a newly defined subcortical FLAIR signal drop (scFLAIR-D) on conventional MRI improve presurgical discrimination between astrocytoma, IDH-mutant (AST) and oligodendroglioma, IDH-mutant and 1p/19q-codeleted (ODG).

**Materials and methods:**

In this single-center retrospective study (January 2021–July 2025), adults with IDH-mutant adult-type diffuse glioma and preoperative T2-weighted and FLAIR MRI were included. Two blinded readers (with senior adjudication) scored five features: redefined T2FM (includes partial but excludes subcortical-only mismatch), scFLAIR-D (focal FLAIR hypointensity confined to subcortical white matter within T2-hyperintensity), multiple necrotic/cystic foci (≥ 2 cavities), cortical predominance (> 50% cortical volume), and calcification (CT).

**Results:**

Forty-one patients were included (median age 42 years; 24 men; 16 AST, 25 ODG). AST significantly more often showed redefined T2FM (AST: 87.5% [14/16] vs. ODG: 20.0% [5/25]; *p* < 0.001) than ODG. ODG significantly more often showed scFLAIR-D (AST: 6.3% [1/16] vs. ODG: 44.0% [11/25]; *p* = 0.013), multiple necrotic/cystic foci (12.5% [2/16] vs. 56.0% [14/25]; *p* = 0.008), and calcification (0% [0/16] vs. 50.0% [12/24 patients with preoperative CT]; *p* < 0.001); cortical predominance trended higher (31.3% [5/16] vs. 64.0% [16/25]; *p* = 0.058). For predicting AST, redefined T2FM achieved 87.5% sensitivity and 80.0% specificity. For predicting ODG, scFLAIR-D achieved 44.0% sensitivity and 93.8% specificity. Two or more ODG-favored features (scFLAIR-D, multiple necrotic/cystic foci, calcification) identified ODG with 100% specificity, whereas redefined T2FM without ODG-favored features identified AST with 96.0% specificity.

**Conclusion:**

A refined T2FM definition improves sensitivity for AST while preserving high specificity. scFLAIR-D serves as a simple, specific marker of ODG; in combination, these routine features yield high rule-in specificity to support presurgical planning and early therapeutic counseling.

**Secondary abstract:**

Refining T2FM to include partial but exclude subcortical-only mismatch increased sensitivity for astrocytoma, IDH-mutant (87.5%) while preserving specificity (80.0%). Newly defined scFLAIR-D was highly specific for oligodendroglioma, IDH-mutant and 1p/19q-codeleted (93.8%). Combining these qualitative MRI/CT features yielded high rule-in specificities for lineage discrimination.

**Supplementary Information:**

The online version contains supplementary material available at 10.1007/s11604-026-01965-z.

## Introduction

The 2021 World Health Organization Classification of Tumors of the Central Nervous System (WHO CNS5) defines adult-type diffuse gliomas by integrated molecular features as astrocytoma, IDH-mutant (AST); oligodendroglioma, IDH-mutant and 1p/19q-codeleted (ODG); and glioblastoma, IDH-wildtype [1, 2]. This taxonomy is clinically actionable: while maximal safe resection is foundational, AST and ODG differ in their natural history and adjuvant treatment paradigms, making reliable preoperative discrimination on MRI and CT directly relevant to surgical goals, adjuvant selection, and patient counseling. Authoritative radiology and neuro-oncology summaries consistently note that ODG carries a more favorable prognosis than AST within IDH-mutant gliomas, aligning expected survival and treatment intensity with lineage [3].

For AST, contemporary guidance supports radiotherapy (RT) followed by adjuvant temozolomide as the reference regimen for CNS WHO grade 3 disease, whereas grade 4 AST is typically managed with a glioblastoma-like backbone of radiotherapy with concurrent and adjuvant temozolomide, individualized by age, performance status, and MGMT promoter methylation [4]. These recommendations reflect a synthesis of randomized and high-quality cohort data and are widely used in practice even when final molecular results lag behind imaging, underscoring the value of a strong preoperative imaging-based probability for AST versus ODG.

By contrast, when ODG (IDH-mutant, 1p/19q-codeleted) requires adjuvant therapy (any grade 3 or high-risk grade 2), the most mature evidence favors radiotherapy followed by PCV (procarbazine, lomustine, vincristine). The joint final analysis of EORTC 26951 and RTOG 9402—with nearly two decades of follow-up—demonstrated durable overall-survival advantages for adding PCV, with the largest benefit in 1p/19q-codeleted tumors, cementing PCV as the preferred alkylator regimen for fit patients [5]. Real-world comparative data further suggest that RT + PCV outperforms RT+temozolomide in newly diagnosed grade 3 ODG, informing consent, logistics, and toxicity trade-offs when ODG is suspected preoperatively [6]. Beyond therapy, recent large-scale analyses highlight lineage-specific prognostic drivers: postoperative tumor volume (a surrogate for extent of resection) shows a stronger, independent association with survival in AST than in ODG, whereas age and histologic grade exert clearer effects in ODG [7]. Therefore, accurate preoperative estimation of tumor lineage may support surgical planning (including extent-of-resection goals and intraoperative mapping strategy) and early counseling, while definitive treatment decisions require integrated molecular diagnosis.

The T2-fluid-attenuated inversion recovery (FLAIR) mismatch sign (T2FM)—defined by near-complete T2-weighted hyperintensity with relative FLAIR hypointensity—is highly specific for AST but has limited sensitivity, motivating exploration of “partial” T2FM and complementary features [8–13]. ODG, in contrast, often demonstrates cortical/subcortical predominance, calcification, and internal heterogeneity, often accompanied by necrosis/cystic change, which may mimic partial T2FM on conventional MRI [14–16]. These well-recognized patterns frame the clinical need for reproducible, lineage-linked imaging biomarkers that can be recognized on routine sequences.

Accordingly, we introduce the subcortical FLAIR signal drop (scFLAIR-D), an intratumoral pattern intended to distinguish intratumoral heterogeneity of ODG from the T2FM phenotype typical of AST. We evaluate the diagnostic contribution of conventional imaging features, including newly defined T2FM (redefined T2FM) and scFLAIR-D, for differentiating IDH-mutant adult-type diffuse gliomas, with the overarching goal of improving preoperative decision-making in light of the lineage-specific prognostic and therapeutic pathways summarized above.

## Materials and methods

This retrospective study received institutional review board approval with a waiver of informed consent; data handling complied with the Health Insurance Portability and Accountability Act, and all records were de-identified before analysis.

### Participants

**Fig. 1:**
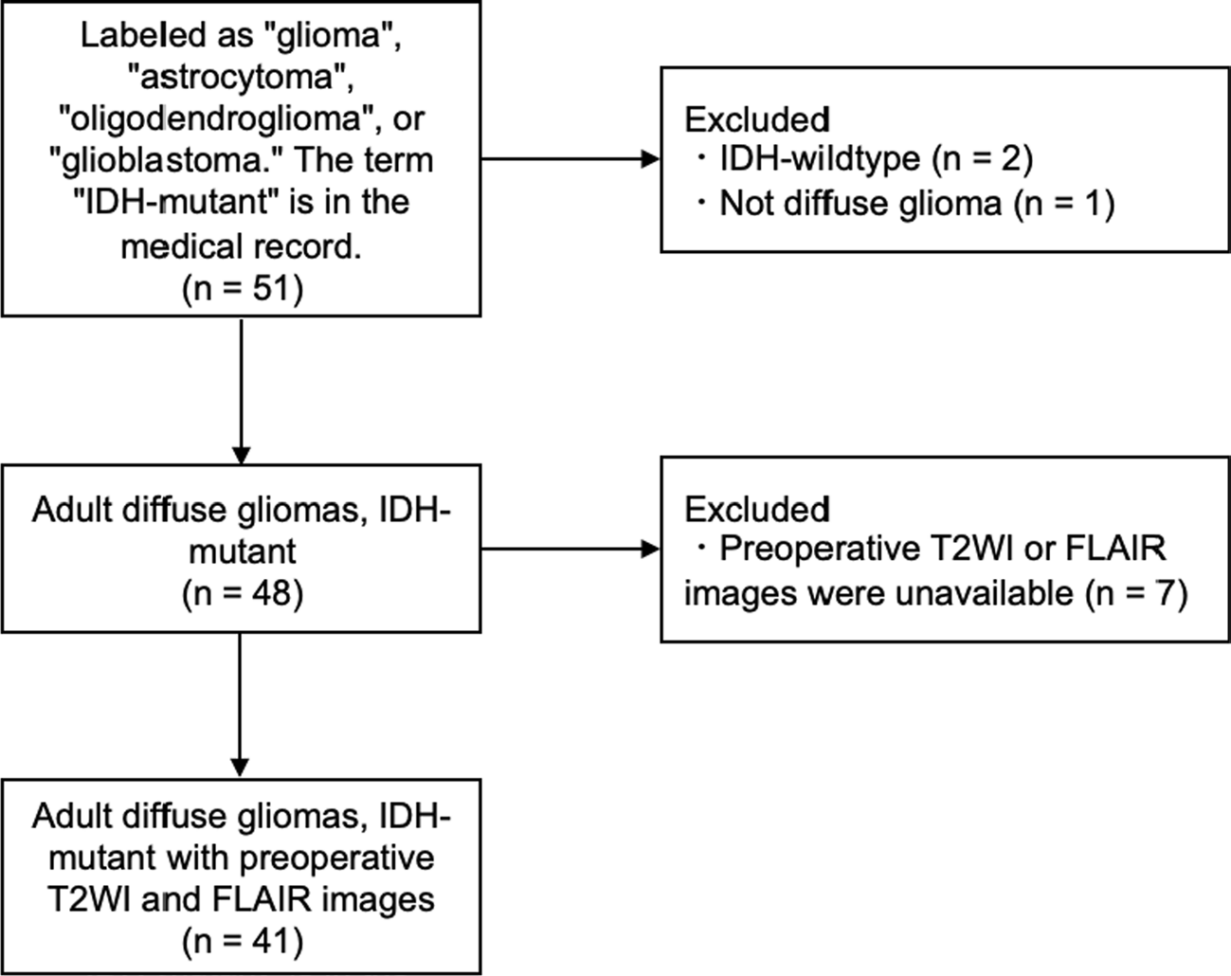
Flow diagram of the patient inclusion and exclusion. T2WI = T2-weighted imaging; FLAIR = fluid-attenuated inversion recovery

We queried the institutional electronic medical record for adults (age ≥ 18 years) registered between January 2021 and July 2025 with diagnostic terms “glioma,” “astrocytoma,” “oligodendroglioma,” or “glioblastoma,” and text evidence of “IDH-mutant.” Patients without newly diagnosed IDH-mutant adult-type diffuse glioma according to the WHO CNS5 diagnostic criteria or lacking preoperative T2-weighted or FLAIR MRI were excluded. A study flowchart details inclusion and exclusion (Fig. 1).

## Image analysis

**Fig. 2:**
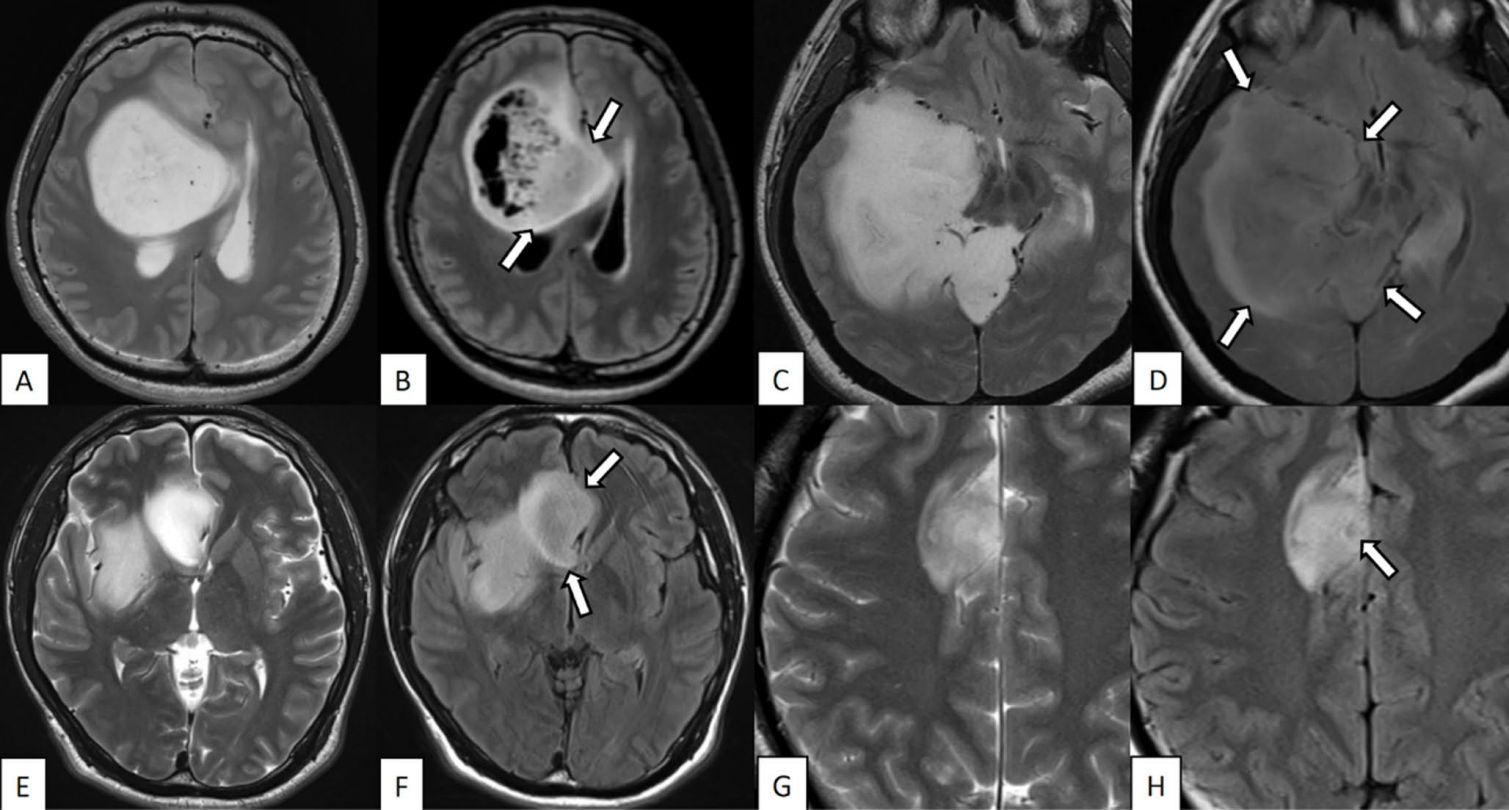
Example cases with positive redefined T2-fluid-attenuated inversion recovery (FLAIR) mismatch sign (T2FM) in astrocytoma, IDH-mutant (AST) and oligodendroglioma, IDH-mutant and 1p/19q-codeleted (ODG). T2-weighted images (**A**,** C**,** E**,** G**) and FLAIR images (**B**,** D**,** F**,** H**) are demonstrated in each case (**A** and** B**: 40 year-old man, AST, CNS WHO grade 3;** C** and** D**: 26 year-old woman, AST, CNS WHO grade 3;** E** and** F**: 26 year-old man, AST, CNS WHO grade 2;** G** and** H**: 32 year-old man, ODG, CNS WHO grade 2). Redefined T2FM is shown in each case; FLAIR-hypointense signal corresponding to T2-hyperintense signal is observed (arrows)

**Fig. 3:**
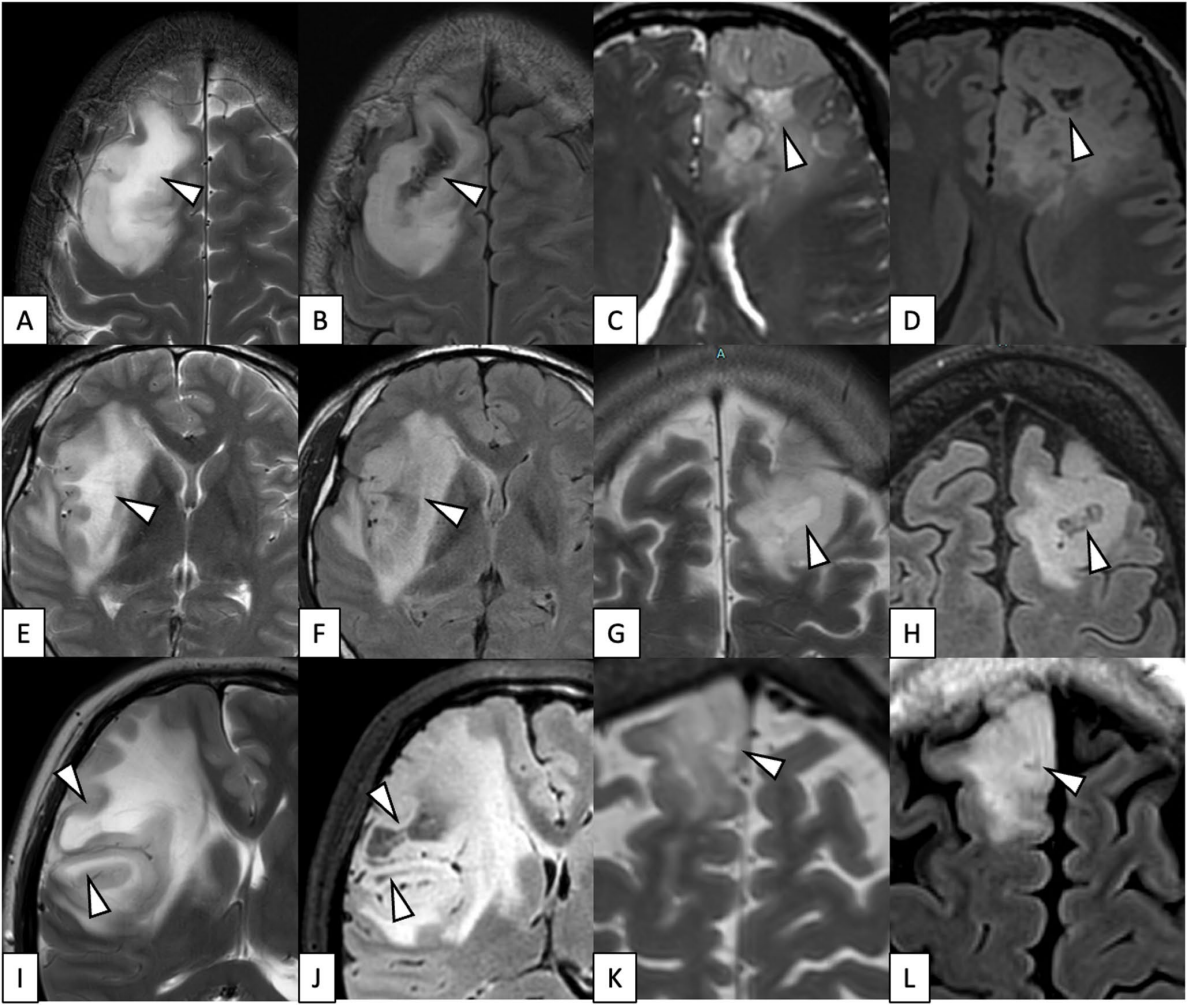
Example cases of subcortical fluid-attenuated inversion recovery (FLAIR) signal drop (scFLAIR-D) in oligodendroglioma, IDH-mutant and 1p/19q-codeleted. T2-weighted images (**A**,** C**,** E**,** G**,** I**,** K**) and FLAIR images (**B**,** D**,** F**,** H**,** J**,** L**) are demonstrated in each case (**A** and** B**: 33 year-old woman, CNS WHO grade 3;** C** and** D**: 44 year-old woman, CNS WHO grade 3; **E** and **F**: 50 year-old man, CNS WHO grade 3;** G** and** H**: 35 year-old woman, CNS WHO grade 3;** I** and** J**: 31 year-old woman, CNS WHO grade 2;** K** and** L**: 56 year-old woman, CNS WHO grade 3). FLAIR-hypointense signal corresponding to T2-hyperintense signal with FLAIR signal drop is observed in the subcortical area (arrow heads)

Preoperative brain MRI (all patients) and non-contrast CT (all but one) were independently reviewed by a trainee radiologist and a board-certified diagnostic radiologist, both blinded to the final diagnosis; disagreements were adjudicated by a senior neuroradiologist who was also blinded. Readers assessed five prespecified features: redefined T2FM, scFLAIR-D, multiple necrotic/cystic foci, cortical predominance, and calcification. Redefined T2FM was considered positive when a tumor region was hyperintense on T2WI and relatively hypointense on FLAIR (Fig. 2). Lesions with T2–FLAIR mismatch confined to the subcortical white matter were not counted as redefined T2FM but as scFLAIR-D. scFLAIR-D was scored when focal FLAIR hypointensity only occurred within T2-hyperintense subcortical white matter, irrespective of degree (Fig. 3). “Multiple necrotic/cystic foci” required ≥ 2 discrete cavities; “cortical predominance” required > 50% of tumor within cortex; calcification was determined on non-contrast CT, given its superiority to MRI for detecting intracranial calcification [17].

### Statistical analysis

Feature frequencies between AST and ODG were compared with Fisher’s exact test (two-sided α = 0.05). Exact binomial 95% confidence intervals were calculated for diagnostic performance metrics. The unweighted Cohen’s kappa (κ) coefficient was calculated to assess interobserver agreement between the two readers. All analyses used R (v4.1.1; R Foundation for Statistical Computing, Vienna, Austria).

## Results

### Characteristics of patients

Forty-one patients were included (24 men, 17 women; median age 42 years [interquartile range: 34–50]); 16 had AST and 25 had ODG (Table 1). All underwent preoperative MRI; 40/41 had non-contrast CT.

**Table 1:** Characteristics of patients and comparison of imaging features and feature combinations

Variable	AST	ODG	P-value
Number	16	25	
Female	5 (31.2%)	12 (48.0%)	
Median age [Interquartile range]	40 [34–47]	44 [35–50]	
CNS WHO grade 2/3/4	6/8/2	12/13/-	
Redefined T2FM	14 (87.5%)	5 (20.0%)	< 0.001*
scFLAIR-D	1 (6.3%)	11 (44.0%)	0.013*
Multiple necrotic/cystic foci	2 (12.5%)	14 (56.0%)	0.008*
Cortical predominance	5 (31.3%)	16 (64.0%)	0.058
Calcification	0 (0%)	12 (50%)**	< 0.001*
Two or more ODG-favored features	0 (0%)	12 (48.0%)	< 0.001*
Redefined T2FM without ODG-favored features	12 (75.0%)	1 (4.0%)	< 0.001*

^*^Statistically significant

^**^Preoperative computed tomography was not performed in one patient

*AST* = astrocytoma, IDH-mutant;* ODG* = oligodendroglioma, IDH-mutant and 1p/19q-codeleted;* CNS* = central nervous system;* WHO* = World Health Organization; *T2FM *= T2-Fluid-attenuated inversion recovery (FLAIR) mismatch sign;* scFLAIR-D* = subcortical FLAIR signal drop

### Feature frequencies in AST and ODG

The results of each imaging feature frequency are displayed in Table 1. Redefined T2FM occurred significantly more often in AST than ODG (AST 14/16 [87.5%] vs. ODG 5/25 [20.0%], *p* < 0.001). scFLAIR-D (1/16 [6.3%] vs. 11/25 [44.0%], *p* = 0.013), multiple necrotic/cystic foci (2/16 [12.5%] vs. 14/25 [56.0%], *p* = 0.008), and calcification (0/16 [0%] vs. 12/24 [50.0%], *p* < 0.001; one ODG lacked CT) were significantly more frequent in ODG. Cortical predominance was numerically higher in ODG (AST 5/16 [31.3%] vs. ODG 16/25 [64.0%], *p* = 0.058).

## Unweighted Cohen’s kappa coefficient

The unweighted Cohen’s kappa coefficients of reader 1 (board‑certified diagnostic radiologist) and reader 2 (trainee radiologist) for each factor were 0.65 (redefined T2FM), 0.29 (scFLAIR-D), 0.79 (Multiple necrotic/cystic foci), 0.71 (Cortical predominance), and 0.88 (Calcification).

In addition, the unweighted κ values for redefined T2FM and scFLAIR-D of reader 1 and reader 3 (a senior neuroradiologist and the final adjudicator) were 0.90 and 0.75, while those of reader 2 and reader 3 were 0.75 and 0.61.

## Diagnostic performance of individual imaging feature and feature combinations

Table 2 shows the diagnostic performance of individual imaging feature and feature combinations.

**Table 2:** Diagnostic performance of imaging features and feature combinations

	Sensitivity	Specificity	Positive predictive value	Negative predictive value	Accuracy
Redefined T2FM	87.5 [64.0–96.5]	80.0 [60.9–91.1]	73.7 [51.2–88.2]	90.9 [72.2–97.5]	82.9 [68.7–91.5]
scFLAIR-D	44.0 [26.7–62.9]	93.8 [71.7–98.9]	91.7 [64.6–98.5]	51.7 [34.4–68.6]	63.4 [48.1–76.4]
Multiple necrotic/cystic foci	56.0 [37.1–73.3]	87.5 [64.0–96.5]	87.5 [64.0–96.5]	56.0 [37.1–73.3]	68.3 [53.0–80.4]
Cortical predominance	64.0 [44.5–79.8]	68.8 [44.4–85.8]	76.2 [54.9–89.4]	55.0 [34.2–74.2]	65.9 [50.5–78.4]
Calcification	50.0 [31.4–68.6]	100 [80.6–100]	100 [75.8–100]	57.1 [39.1–73.5]	70.0 [54.6–81.9]
Two or more ODG-favored features	48.0 [30.0–66.5]	100 [80.6–100]	100 [75.8–100]	55.1 [37.5–71.6]	68.3 [53.0–80.4]
Redefined T2FM without ODG-favored features	75.0 [50.5–89.8]	96.0 [80.5–99.3]	92.3 [66.7–98.6]	85.7 [68.5–94.3]	87.8 [74.5–94.7]

Data are presented as percentages with 95% confidence intervals

*T2FM* = T2-fluid-attenuated inversion recovery (FLAIR) mismatch sign; scFLAIR-D = subcortical FLAIR signal drop

For predicting AST, redefined T2FM yielded sensitivity 87.5% and specificity 80.0%. For predicting ODG, scFLAIR-D yielded sensitivity 44.0% and specificity 93.8%.

Presence of ≥ 2 ODG-favored features (scFLAIR-D, multiple necrotic/cystic foci, calcification) suggested ODG with sensitivity 48.0% and specificity 100%. Redefined T2FM without any ODG-favored features suggested AST with sensitivity 75.0% and specificity 96.0%.

## Discussion

We showed that a refined definition of T2FM—permitting partial positivity but excluding subcortical-only mismatch—preserves high specificity for AST while improving sensitivity relative to classic, near-complete T2FM.

In this study, we evaluated the diagnostic performance of conventional MRI and non-contrast CT in differentiating IDH-mutant adult-type diffuse gliomas. Redefined T2FM preserves high specificity (80.0%) for AST while improving sensitivity (87.5%) relative to classic, near-complete T2FM. Furthermore, we found that scFLAIR-D—a focal subcortical FLAIR signal drop within T2-hyperintensity—was significantly more frequent in ODG and achieved 93.8% specificity for ODG, likely reflecting contributions from cystic/necrotic components and microstructural fluid spaces that attenuate FLAIR signal [18, 19]. We also identified the following two practical criteria to differentiate AST and ODG: “One or more ODG-favored features (scFLAIR-D, multiple necrotic/cystic foci, and calcification) suggest ODG”(sensitivity of 48.0%, specificity of 100% [95% confidence interval (95% CI): 75.8–100]) and “redefined T2FM without ODG-favored features suggest AST”(sensitivity of 75.0%, specificity of 96.0%).

T2FM was first reported as an imaging finding observed exclusively in IDH-mutant, 1p/19q-non-codeleted glioma (i.e., AST), and not in IDH-mutant, 1p/19q-codeleted glioma (i.e., ODG) or IDH-wildtype glioma [20]. T2FM is considered to reflect microcystic changes and enlarged intercellular spaces of the tumors [21–23]. A meta-analysis study confirmed sensitivity and specificity of T2FM in differentiating AST from other types of gliomas as 40% and 100%, respectively [8]. The generally accepted definition of the conventional T2FM is “a complete or near-complete hyperintense signal on T2WI with a relatively hypointense signal on FLAIR except for a hyperintense peripheral rim, excluding areas of necrosis or cystic change” [20, 24]. Although the complete/near-complete T2FM has demonstrated very high specificity for distinguishing between AST and ODG, the low sensitivity has posed a clinical problem [8–11, 25, 26]. Cho et al. [27] reported that lowering the threshold for the percentage of T2FM volume could improve the sensitivity for detecting AST. Partial T2FM, which is considered positive if the mismatch is observed in only a part of the tumor, was confirmed to be highly specific for IDH-mutation in WHO grade 4 gliomas, and more frequent in AST than in the complete/near-complete T2FM [12, 13]. The present study validated these results.

On the other hand, calcification, frequent cortical–subcortical involvement, necrosis or cystic change have been well-known imaging features of ODG [14–16]. These characteristics were also validated in the present study. Furthermore, we newly identified that scFLAIR-D predicted ODG with a high specificity of 93.8%. In this study, T2FM was redefined by excluding scFLAIR-D based on intratumoral distribution. We assume that redefined T2FM and scFLAIR-D share microstructural contributors that attenuate FLAIR signal (e.g., microcystic change and enlarged intercellular spaces). However, when the definition is broadened to include partial T2FM, heterogeneous cavities that may represent cystic degeneration or necrosis can be inadvertently included. By classifying subcortical-dominant cavities and related heterogeneity as scFLAIR-D, we aimed to increase the ‘purity’ of redefined T2FM as a closer surrogate of the classic T2FM microcystic phenotype.

In order to make these findings more applicable to real-world imaging diagnosis, we conducted an analysis of the diagnostic value of feature combination. We found that two or more ODG-favored features predicted ODG with 100% specificity [95% CI: 75.8–100], and that redefined T2FM without ODG-favored features predicted AST with 96.0% specificity.

In this study, the unweighted Cohen’s kappa of redefined T2FM and scFLAIR-D between reader 1 and reader 2 were 0.65 and 0.29, which were relatively low. However, the inter-reader agreement between Reader 1 and Reader 3 was generally higher than that between Reader 2 and Reader 3. This suggests that interpretation of imaging features of scFLAIR-D and redefined T2FM requires a certain level of experience, and that discrepancies in assessment are more likely to occur between trainees and experienced diagnostic radiologists.

Furthermore, this study was deliberately designed to address a focused question: whether routine MRI and non-contrast CT can provide reproducible qualitative features for lineage discrimination between AST and ODG within IDH-mutant adult-type diffuse gliomas. Restricting the cohort to IDH-mutant tumors minimized biological and imaging confounders typical of IDH-wildtype glioblastoma, allowing assessment of scFLAIR-D and redefined T2FM as a lineage-associated rather than grade-related feature. However, this selection limits the real-world applicability of this study, and future targeted validation studies are warranted.

The present study has several limitations. First, this was a single-center retrospective study with a modest sample size; therefore, multivariable modeling was underpowered and should be interpreted cautiously. Second, imaging protocols were not standardized across scanners because of the retrospective nature (Supplementary Data). Therefore, validation through multicenter studies using a standardized protocol is desirable. Third, due to heterogeneity in imaging conditions, quantitative assessments were not performed. However, the use of qualitative assessments, which radiologists widely employ in routine clinical practice, enhances the clinical applicability of our approach. Fourth, because of the retrospective design, tumors were resected without neuronavigation-based identification or targeted sampling of the scFLAIR-D region. Although we discuss plausible mechanistic bases for scFLAIR-D, direct radiologic-pathologic correlation could not be performed. Also, other imaging features, such as multiple necrotic/cystic foci, are not histopathologically confirmed. Therefore, prospective studies with spatially matched tissue sampling are warranted. Finally, the present study did not include comparisons with IDH-wildtype gliomas, including pediatric-type diffuse gliomas, which may also show T2FM [28–30]. This fact limits the real-world applicability of this study. Future studies incorporating these tumor types, especially IDH-wild type diffuse glioma, are warranted.

## Conclusion

Including partial cases and excluding subcortical cases, redefined T2FM predicted AST with 87.5% sensitivity while maintaining 80.0% specificity. scFLAIR-D predicted ODG with 93.8% specificity. Two or more ODG-favored features (scFLAIR-D, multiple necrotic/cystic foci, or calcification) suggested ODG with 100% specificity [95% CI: 75.8–100], and redefined T2FM without ODG-favored features suggested AST with 96.0% specificity.

## Supplementary Information

Below is the link to the electronic supplementary material.

Supplementary Material 1

## Data Availability

The data that support the findings of this study are available from the corresponding author upon reasonable request.

## Abbreviations

AST: Astrocytoma, IDH-mutant
FLAIR: Fluid-attenuated inversion recovery
ODG: Oligodendroglioma, IDH-mutant and 1p/19q-codeleted
scFLAIR-D: Subcortical FLAIR signal drop
T2FM: T2-FLAIR mismatch sign
