# Supplementary material for: Conventional MRI/CT for differentiation of IDH-mutant adult-type diffuse gliomas: revisited with a new imaging feature “scFLAIR-D”
Source: Jpn J Radiol. 2026 Mar 27;44(7):1142–9. doi: 10.1007/s11604-026-01965-z (PMC13315118; doi:10.1007/s11604-026-01965-z)
Supplement: Supplementary file 1 — Supplementary Material 1 [file 11604_2026_1965_MOESM1_ESM.pdf]

Supplementary data

Table 1. Number of acquisition conditions

| Sequence | Field strength (T) | 2D acquisition | 3D acquisition |
|---|---|---|---|
| T2WI | 1.5 | 7 | 0 |
| | 3 | 30 | 4 |
| FLAIR | 1.5 | 7 | 0 |
| | 3 | 31 | 3 |

The number of 2D and 3D acquisitions for each magnetic field strength (1.5 T or 3 T) and sequence (T2WI or FLAIR) is shown.

2D = two dimensions; 3D = three dimensions; T2WI = T2-weighted imaging; FLAIR = fluid-attenuated inversion recovery

Table 2. MRI acquisition parameters in each conditions

| | 2D T2WI | 3D T2WI | 2D FLAIR | 3D FLAIR |
|---|---|---|---|---|
| Repetition time (ms) | 4359 [4000–4460] | 2500 [2375–2675] | 9002 [9000–10000] | 5000 [4900–6500] |
| Echo Time (ms) | 84.54 [83.14–91] | 317.90 [312.58–340.52] | 117.91 [108.50–119.65] | 265.32 [247.19–398.16] |
| Inversion Time (ms) | | | 2473.19 [2412.50–2640.00] | 4361 [4000–4460] |
| Slice Thickness (mm) | 3 [3–5] | 1.70 [1.45–1.85] | 5 [5–5] | 1.00 [0.95–1.3] |
| Rows | 512 [512–512] | 496 [480–512] | 512 [480–512] | 640 [576–704] |
| Columns | 512 [464–512] | 496 [480–512] | 512 [468–512] | 640 [576–704] |
| FOV (mm) | 210.02 [210.00–220.00] | 248.00 [240.00–256.00] | 210.02 [210.00–227.49] | 240.00 [235.00–248.00] |

Data are median and interquartile range.

*The length of one side of FOV is shown.

2D = two dimensions; 3D = three dimensions; T2WI = T2-weighted imaging; FLAIR = fluid-attenuated inversion recovery; FOV = field of view
